# Plasma Amyloid‐β Levels are Associated with Risk of Heart Failure: Framingham Heart Study

**DOI:** 10.1002/alz70856_106223

**Published:** 2026-01-08

**Authors:** Dibya Himali, Jeremy A. Tanner, Alexa S Beiser, Matthew P. Pase, Emer McGrath, Vasileios‐Arsenios Lioutas, Vasan S. Ramachandran, Jose Rafael Romero, Sudha Seshadri, Jayandra Jung Himali, Hugo J. Aparicio

**Affiliations:** ^1^ The Framingham Heart Study, Framingham, MA, USA; ^2^ Boston University School of Medicine, Boston, MA, USA; ^3^ University of Texas Health San Antonio, San Antonio, TX, USA; ^4^ Department of Biostatistics, Boston University School of Public Health, Boston, MA, USA; ^5^ Department of Neurology, Boston University Chobanian & Avedisian School of Medicine, Boston, MA, USA; ^6^ Monash University, Clayton, VIC, Australia; ^7^ University of Galway, Galway, Galway, Ireland; ^8^ Beth Israel Deaconess Medical Center, Boston, MA, USA; ^9^ The University of Texas School of Public Health in San Antonio, San Antonio, TX, USA; ^10^ The Long School of Medicine, University of Texas Health Science Center, San Antonio, TX, USA; ^11^ Framingham Heart Study, Framingham, Boston, MA, USA; ^12^ Boston University Chobanian & Avedisian School of Medicine, Boston, MA, USA; ^13^ South Texas Alzheimer's Disease Research Center, San Antonio, TX, USA; ^14^ Department of Neurology, UT Health San Antonio, 7703 Floyd Curl Drive, San Antonio, TX, USA; ^15^ Glenn Biggs Institute for Alzheimer's & Neurodegenerative Diseases, University of Texas Health Sciences Center at San Antonio, San Antonio, TX, USA; ^16^ Graduate School of Biomedical Sciences, University of Texas Health Science Center, San Antonio, TX, USA; ^17^ Glenn Biggs Institute for Alzheimer's & Neurodegenerative Diseases, University of Texas Health San Antonio, San Antonio, TX, USA; ^18^ Department of Population Health Sciences, UT Health San Antonio, San Antonio, TX, USA

## Abstract

**Background:**

Amyloid‐β (Aβ) is a toxic polypeptide of 36‐43 amino acids formed during proteolysis. Initial studies suggest that protein misfolding, including abnormal aggregation of Aβ, is associated with cardiac dysfunction. Our objective is to investigate the association of circulating plasma amyloid‐β40 (Aβ40), amyloid‐β42 (Aβ42), and Aβ42/40 with the incidence of congestive heart failure (HF).

**Method:**

We evaluated the association of plasma Aβ40, Aβ42, and Aβ42/40 with risk of HF in a community‐based sample from the prospective Framingham Heart Study. We included adult participants aged 45+ years, free from clinical HF at the time of blood draw (Offspring cohort, Exam 7, 1998‐2001). Plasma Aβ40 and Aβ42 were measured using the Fluorometric immunoassay platform. Aβ levels were natural log‐transformed to normalize the distribution. Using Cox regression models, each of these biomarkers were related to incident HF, adjusting for age and sex. Previous studies identified differences in the association of Aβ with HF by sex, so we tested for interaction by sex and reported stratified results when statistically significant (a=0.05).

**Result:**

Among the 3,125 included participants (mean±SD age 62.1±8.9 years, 54% women), there were 146 (4.7%) incident HF events over 10 years of follow up. Higher plasma Aβ40 levels (per unit of the natural log of the protein level) were associated with an increased risk of HF (hazard ratio [HR] 95% confidence interval [CI], p): (2.04[1.03‐4.02], 0.040) but not with Aβ42 (1.48[0.74‐2.98], 0.273) and Aβ42/40 (0.73[0.37‐1.44], 0.365). We observed effect modification by sex in the association between each of the Aβ40 (*p* = 0.032), Aβ42 (*p* = 0.027) and risk of HF but not with Aβ42/40 (*p* = 0.867). Stratified analyses revealed that Aβ40 and Aβ42 levels were associated with an increased risk of HF in men (HR 95%[CI], p): 4.50 [1.67‐12.11], 0.003) and (3.14 [1.19‐8.27], 0.021), respectively but not in women (0.96 [0.37, 2.53], 0.937) and (0.64 [0.24, 1.73], 0.381), respectively.

**Conclusion:**

Elevated plasma levels of Aβ40 and Aβ42 were associated with higher risk of heart failure, in men only. These results provide further evidence that circulating proteins in the blood can provide prognostic information on diseases affecting the brain‐heart axis, possibly linked by protein misfolding or aggregation.

